# The satisfaction regarding handovers between ambulance and emergency department nurses: an observational study

**DOI:** 10.1186/s13049-018-0545-7

**Published:** 2018-09-10

**Authors:** Gijs Thomas Hovenkamp, Tycho Joan Olgers, Remco Robert Wortel, Milou Esmée Noltes, Bert Dercksen, Jan Cornelis ter Maaten

**Affiliations:** Department of Internal Medicine, Emergency Medicine, University of Groningen, University Medical Center Groningen, Hanzeplein 1 9700RB, Groningen, The Netherlands

**Keywords:** Acute care, Ambulance nurse, ED nurse, Emergency department, Handoff, Handover, Satisfaction, Transfer

## Abstract

**Background:**

A thorough handover in the emergency department (ED) is of great importance for improving the quality and safety in the chain of care. The satisfaction of handover may reflect the quality of handover. Research to discover the variables influencing the satisfaction of handovers is scarce. The goal of this study was to determine the factors influencing the satisfaction regarding handovers from ambulance and ED nurses.

**Methods:**

We performed a prospective observational study in the University Medical Center of Groningen. Data regarding prehospital-hospital handovers has been collected by observing handovers and assessing patient chart information. Data regarding the satisfaction has been collected with a questionnaire including a 5-point scale for the level of satisfaction.

**Results:**

In total, 97 handovers were observed and 97 ambulance nurses and 89 ED nurses completed the questionnaire. The satisfaction of ambulance nurses showed a negative correlation with the waiting time prior to handover (*r =* −.287, *p* = .004) and a positive correlation with the presence of a physician in the receiving team (*r =* .224, *p =* .028). The satisfaction of ED nurses showed a positive correlation with the use of the ABCDE (*r =* .288, *p* = .006) and AMPLE instrument (*r =* .208, *p* = .050).

**Conclusion:**

The satisfaction of ambulance and ED nurses as sender or receiver of the handover is determined by different factors. The satisfaction of ambulance nurses is mainly affected by the waiting time and presence of a physician, while the satisfaction of ED nurses is affected by the use of handover instruments and the completeness of medical information.

**Electronic supplementary material:**

The online version of this article (10.1186/s13049-018-0545-7) contains supplementary material, which is available to authorized users.

## Background

A thorough handover in the emergency department is of great importance for improving the quality and safety in the chain of care. A good handover is associated with an improvement in patient safety, as well as continuity of patient care and improved decision making [1–6]. Most research regarding clinical handovers has focused on nurse-nurse or physician-physician handovers but only a small amount of research actively focuses on pre-hospital (ambulance) to ED nurses [7–13]. Several qualitative studies showed that active listening and the use of a handover instrument improved the quality of a handover while distractions did not have any effect on the quality [6, 14–17]. However, these studies did not have a standardized method of measuring quality. The satisfaction of handover may therefore reflect the quality of care. An unstructured or incomplete handover can be annoying and may influence active listening risking loss of information. The variables influencing this satisfaction are unknown.

Recent studies show that improving nurses’ job satisfaction improves quality of care and patient safety [18, 19]. Furthermore, it is known that better work environments are associated with higher quality of care and also higher patient satisfaction [20]. Research suggests that variables influencing handover satisfaction differ between the several parties (prehospital vs. hospital personnel and handing over vs. receiving). For example, it is frustrating for ambulance nurses if the receiving ED team is already taking care of the patient during the handover instead of first listening to the handover [6, 21, 22]. Research also suggests there are difficulties in creating a shared cognitive picture between ambulance and ED nurses [6]. It also seems that a frequent source of frustration of the receiving team during handovers is caused by insufficient and incomplete medical information [6, 10]. Because these are all qualitative studies and there is still no quantitative study on this subject, we decided to perform a quantitative study.

Since improving the work environment and satisfaction results in an improved quality of care and patient safety, this may also be true for the satisfaction of handover. The goal of this study is to discover which variables influence the satisfaction of the handover for ambulance and ED nurses.

## Methods

### Study design

We performed a prospective observational study regarding handovers from ambulance to ED nurses.

### Study stetting and population

This research was conducted in the ED of the University Medical Center Groningen (UMCG), a tertiary care teaching hospital with over 34,000 visits to the ED annually. Data were collected two days a week from March till July 2016 from 10:00 till 17:00, varying from Monday to Friday, based on the availability of the observers. Patients were included if they were 18 years or older and if they were admitted by ambulance. Exclusion criteria were any kind of traumatic injuries, since trauma-handovers are already standardized in the UMCG. In other cases, there is no predefined structured handover. There are, however, unwritten expectations to use one of the three common handover instruments in the Netherlands; the SBAR (Situation, Background, Assessment, Recommendation), AMPLE (Allergies, Medicine, Past, Last meal, Event) or ABCDE (Airway, Breathing, Circulation, Disability, Exposure) instrument [23, 24].

Following informed consent from ambulance nurses, ED nurses and the patient, data collection was done by four trained medical students and included assessment of patient chart information, observing handovers and collecting questionnaires. These questionnaires were filled in by the ambulance and ED nurses. Since a regular ambulance team in the Netherlands consists of one driver and one ambulance nurse, we paid extra attention that the questionnaire was filled in by the nurse, since the drivers have less medical expertise and did not have the same education. Data obtained from patient records were anonymously stored using study-specific patient codes in a password protected database.

### Training

Data were collected by four medical students. To reduce interobserver variation, the students were trained by two experienced ED physicians (supervisors) which included role-plays and observing real handovers with the supervisors. After each role-play or observed handover, the Case Report Form (CRFs) were compared and conflicting results were discussed, with great attention to the use of handover instruments, since these are difficult to objectify. Handover instruments were scored by allocating one point for each used letter, so the ABCDE and AMPLE instrument could get a maximum of 5 points each and the SBAR a maximum of 4 points. Since it can be hard to objectify the usage of mnemonics, the first two days of observing was done in pairs. When a letter was not mentioned specifically, but the medical information was mentioned (e.g. *“there is no sign of a cardiovascular problem” instead of “C: no problem”*) it was interpreted as correct use of the ‘C’ in ABCDE. Although there is a great amount of handover instruments, these three were chosen because they are frequently used and taught in the Netherlands [23, 24].

### Patient chart information

Patient charts were used to collect data regarding gender, age and the triage code. The triage code was allocated by a trained triage nurse based on the main complaint, basic vital parameters and expected amount of resources needed, according to the emergency severity index. There are 5 possible triage codes: red meaning immediate resuscitation needed; orange almost immediate resuscitation needed (within 10 min); yellow means a potentially ill patient with resuscitation needed within 1 h or more resources (investigations/diagnostics) needed; green no resuscitation needed but treatment within 2 h and only one resource needed; blue no resources needed and treatment within 4 h. The patient chart was also used for specific pre-hospital information like estimated time of arrival, reason for referral, administered medication and ambulance urgency, divided in three groups: ‘A1’ meaning there is a life-threatening condition; ‘A2’ meaning there is no immediate life-threatening condition and ‘B’ meaning planned transport and administered medication. Each handover was observed by one student. The structure and content of the handover was registered on the CRF (see Additional file 1).

### Questionnaire

The questionnaire was used for determining the level of overall satisfaction of the handover and factors influencing this satisfaction. We measured the satisfaction with a 5-point scale with the following scoring: 1 = ‘strongly disagree’, 2 = ‘disagree’, 3 = ‘neutral’, 4 = ‘agree’ and 5 = ‘strongly agree’. A list with possible factors influencing the satisfaction for both teams was constructed by a multidisciplinary team, including emergency physicians, internists and the medical directors of the ambulance service. We asked 9 ambulance and 9 ED nurses to review this list and give feedback upon which we constructed the definitive questionnaire. The questionnaires for both parties differed slightly because some factors, e.g. ‘waiting time’, were not considered relevant for both parties, since the receiving nurse has no waiting time prior to handover. The complete questionnaire is available as an additional file (see Additional file 2).

### Statistics

We used IBM SPSS Statistics 23.0 to analyze the collected data. Correlation analysis between satisfaction of ambulance or ED nurses and several factors regarding handover was done using Spearman’s rho. The factors analyzed for correlation with ambulance nurse satisfaction were: duration, interruptions, the presence of a physician, questions to clarify and the waiting time prior to handover. The factors analyzed for correlation with ED nurse satisfaction were: duration, interruptions, the use of handover instruments and years of work experience of the ambulance nurses. A *P*-value of 0.05 or less was considered significant. Data is presented using means ± standard deviation.

## Results

In total, 97 handovers were observed. The baseline characteristics of the study population are shown in Table 1. The questionnaire was completed 97 times by ambulance nurses and 89 times by ED nurses. As shown in Table 2, the overall satisfaction was high in both groups (ambulance nurses 4.2 ± 0.8 and ED nurses 4.0 ± 1.0), 6 ambulance nurses and 9 ED nurses were unsatisfied (score 1 or 2).

**Table 1:** Data of study population

Patients (n)	97
Age (years)	65.0 ± 15.0
Gender (Male/Female)	53/44
Color code assigned by triage nurses*	
Red: n (%)	2 (2.1)
Orange: n (%)	32 (33.0)
Yellow: n (%)	60 (61.9)
Green: n (%)	0 (0)
Blue: n (%)	1 (1.0)
Unknown: n (%)**	2 (2.1)

*Red = very urgent, orange = urgent, yellow = not very urgent, green = regular, blue = not urgent

**The triage code was not registered for 2 patients

**Table 2:** Data of handovers

Amount of completed questionnaires by ambulance nurses	97
Satisfaction ambulance nurses (1–5)	4.2 ± 0.8
Work experience (years)	13.1 ± 8.3
Amount of completed questionnaires by ED nurses	89
Satisfaction ED nurses (1–5)	4.0 ± 1.0
Work experience (years)	10.1 ± 9.9
Composition ED team	
Nurse only(n)	77
Nurse and physician: (n)	19
Physician only: (n)	1
Duration of handover (seconds)	174 ± 73
Handovers including one or more interruption(s) *: (n)	26
Handovers including one or more question(s): (n)	86
Waiting time prior to handover (seconds)	251 ± 198

*Questions to clarify were not considered as an interruption

### Satisfaction of ambulance nurses

The main reasons for ambulance nurses being less satisfied was absence of an ED physician and waiting times (Tables 3 and 4). Ambulance nurses were unsatisfied in 1 of 20 cases if the physician was present and unsatisfied in 5 of 77 handovers if the physician was absent (positive correlation *r =* .224, *p* = .028). In the majority of cases when a doctor was present during handover it concerned a red or orange triaged case (84%). In 46.7% of times when an ambulance nurse thought improvement was needed the reason was the waiting time prior to handover (negative correlation *r =* −.287, *p =* .004). There was no correlation between the emergency severity index and ambulance nurse satisfaction (*r* = −.169, *p* = .101). A final issue was the necessity to perform handover in the hall instead of a private room (5 times a reason for being less satisfied).

**Table 3:** Satisfaction of ambulance nurses

Handover data	Correlation coefficient*	*P*-Value
Duration of handover	.055	.593
Interruptions during handover	.028	.787
Physician present during handover	.224	.028
Questions asked during handover	.006	.956
Waiting time prior to handover	−.287	.004
Work experience ED nurse	−.077	.486

*Spearman’s rho correlation analysis between satisfaction of ambulance nurses and several factors regarding handover

**Table 4:** Number of reasons (not) to be satisfied regarding handover by ambulance nurses (*n* = 97)

	Improvement needed	Satisfied
Composition of ED team	2 (13.3%)	42 (15.4%)
Duration of handover	1 (6.7%)	39 (14.3%)
Interruptions during handover	2 (13.3%)	48 (17.6%)
Questions to clarify	2 (13.3%)	49 (17.9%)
Timing of handover	1 (6.7%)	58 (21.2%)
Waiting time prior to handover	7 (46.7%)	37 (13.6%)
Total	15	273

### Satisfaction of ED nurses

Nine ED nurses were unsatisfied with main reasons being lack of a structured handover instrument, incomplete information or large disagreement between prehospital announcement and patient condition at ED entry. When more letters from the ABCDE or the AMPLE instrument were used, the satisfaction increased (correlation for ABCDE *r =* .288, *p =* .006 and for AMPLE instrument *r* = .208, *p =* .050). In 51.5% of times an ED nurse thought improvement was needed it was regarding the use of a handover instrument (Tables 5 and 6).

**Table 5:** Satisfaction of ED nurses

Handover Data	Correlation coefficient*	*P*-Value
Duration of handover	.151	.159
Interruptions during handover	−.053	.620
Use handover instrument		
ABCDE	.288	.006
AMPLE	.208	.050
SBAR	.131	.222
Work experience ambulance nurse	−.200	.062

*Spearman’s rho correlation analysis between satisfaction of ambulance nurses and several factors regarding handover

**Table 6:** Number of reasons (not) to be satisfied regarding handover by ED nurses (*n* = 89)

	Improvement needed	Satisfied
Complete information received	5 (15.2%)	49 (23.2%)
Duration of handover	1 (3.0%)	28 (13.3%)
Interruptions during handover	4 (12.1%)	39 (18.5%)
Prior information notice	4 (12.1%)	18 (8.5%)
Timing of handover	2 (6.1%)	39 (18.5%)
Use of handover instrument	17 (51.5%)	38 (18.0%)
Total	33	211

**Fig. 1:**
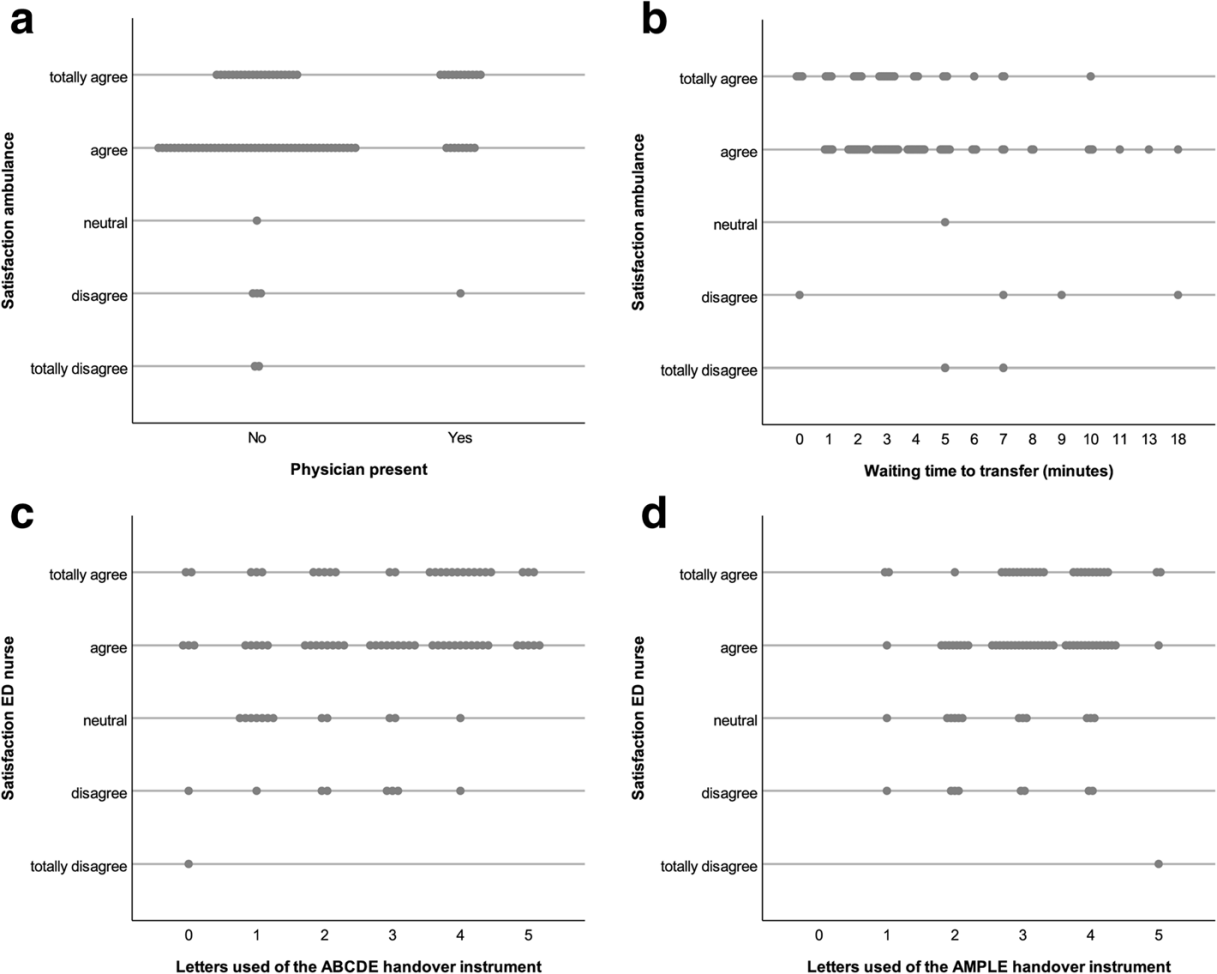
Scatterplots of most important findings

There was a negative correlation (*r* = −.240, *p* = .019) between work experience and the use of the ABCDE instrument. Figure 1 shows the scatterplots for most important findings.

## Discussion

This study shows that ambulance nurses and ED staff are generally satisfied with the handover in our hospital. The presence of a physician during handover and shorter waiting times till handover are important factors for improvement of handover satisfaction for ambulance nurses. The use of handover instruments such as ABCDE or AMPLE is an important factor determining handover satisfaction for the ED nurses. To our knowledge, this is the first quantitative study which focuses on differences between the satisfaction of ambulance and ED nurses regarding handovers.

Our results are in line with a recent study which reported that the overall satisfaction in patient care teams improved after the implementation of a structured handover checklist [25]. In addition, recent qualitative research shows that emergency care providers believe the structure and handover benefit from standardization [26]. Furthermore, other studies show that handover instruments have a positive influence on patient safety [27–29]. We now show that the use of handover instruments with a clear structure also correlates also with a higher handover satisfaction.

This satisfaction might be of importance since nurses seem to be less satisfied with their jobs than physicians and job satisfaction and the perceived work environment correlate with the quality of care and handover safety [30–32]. Furthermore, there is a correlation between years of work experience from ambulance nurses and the use of the ABCDE instrument. We speculate that this might be explained by the fact that less experienced nurses benefit more from certain handover instruments or that they are more frequently taught nowadays.

We realize that this study has several limitations. First, there is the possibility of interobserver variability despite our effort with intensive mutual training to reduce this bias. Second, there was a relative small number of handovers (97), which may cause some factors to be unfairly insignificant. Although the satisfaction of handovers can improve when there is an observer present (Hawthorne effect), we believe this does not change the specific variables influencing the satisfaction. Furthermore, we have addressed several factors of which we thought they might influence satisfaction. Although these were carefully chosen factors by our multidisciplinary team, it is possible that other factors we did not take into account also influence this satisfaction. Finally, we have measured the satisfaction of the handover but not the quality of the handover itself or the exact relation between satisfaction and the quality of the handover and patient care. We think satisfaction may reflect the quality of handover. For example active listening and reducing the risk missing important information could be promoted by a structured handover and increasing satisfaction, but we have not addressed this question in our study. The relation between satisfaction and quality of care would be an important question in subsequent studies.

Since measuring the actual quality of handovers is complicated and there is no clear definition of the quality of handovers, we chose to focus on the satisfaction. While his study gives us new insights in the determinants of the satisfaction regarding handovers, determinants influencing the quality of handover are not yet known. If further research is able to find a way to objectify the quality of handovers, they could focus on the variables influencing this quality instead of the satisfaction. The combination of knowledge of both, the satisfaction and quality, could help improve the complicated process of handovers. With our current knowledge, we advise to integrate handover instruments as the ABCDE and AMPLE in a standardized handover protocol. Further research to objectify the quality of handovers could focus on the use of such a standardized protocol.

## Conclusion

The satisfaction of ambulance and ED nurses as sender or receiver of the handover is determined by different factors. The satisfaction of ambulance nurses is mainly affected by the waiting time and presence of a physician while the satisfaction of ED nurses is mainly affected by the use of handover instruments. This new information gives more insight in the complexity of handing over and could eventually result in an improved quality of care.

## Additional files

Additional file 1: “Case Report Form”, PDF. The standardized form used for collection of data. (PDF 360 kb)

Additional file 2: “Questionnaire”, PDF. The questionnaires used for the measurement of satisfaction regarding the handover. Both teams (handing over and receiving) used a different questionnaire. (PDF 134 kb)

## Abbreviations

ABCDE: Airway, Breathing, Circulation, Disability, Exposure
AMPLE: Allergies, Medication, Past, Last meal, Events
CRF: Case Report File
ED: Emergency Department
SBAR: Situation, Background, Assessment, Recommendation
UMCG: University Medical Centre Groningen
