# Supplementary material for: The satisfaction regarding handovers between ambulance and emergency department nurses: an observational study
Source: Scand J Trauma Resusc Emerg Med. 2018 Sep 10;26:78. doi: 10.1186/s13049-018-0545-7 (PMC6131795; doi:10.1186/s13049-018-0545-7)
Supplement: Supplementary file 1 — “Case Report Form”, PDF. The standardized form used for collection of data. (PDF 360 kb) [file 13049_2018_545_MOESM1_ESM.pdf]

Additional file 1

Patient number

Subject number:

Subject nummer ☐☐☐☐☐☐

Case Report Form

Handover from Ambulance to Emergency Department

G00. General Information

Date screening (day/month/year): ☐☐ / ☐☐ / ☐☐

Data collected by:…………...…………………………………………………...

Verbal informed consent given: Yes ☐ No ☐

Gender: Male ☐ Female ☐

Age: ☐☐

Specialism: ☐☐☐☐

Urgency CPA towards patient: A1 ☐ A2 ☐

Urgency CPA towards ED: A1 ☐ A2 ☐

Triage by ED: Red ☐ Orange ☐ Yellow ☐

Reason of referral: Circulatory ☐ Respiratory ☐ Digestive ☐
Urogenital ☐ Musculoskeletal ☐ Endocrine ☐
Central nerve system ☐ Hematological ☐ Other ☐

G01. Pre-Hospital information

G01-01: Estimated Time of Arrival: ☐☐ : ☐☐

G01-02: Refferer: General Practitioner ☐ CPA ☐ Other hospital ☐

G01-03: Pre-Hospital information received: Yes ☐ No ☐

Subject nummer ☐☐☐☐☐☐

G01-04: Pre-hospital information by: GP ☐ Medical expert ☐ MKA ☐

Ambulance digitally ☐ Ambulance phone ☐

G02. Vital parameters

	First values by ambulance	MEWS	Values during handover	Parameters mentioned during handover	MEWS	First values on the ED	MEWS
Respiratory rate per minute				Yes/No			
O2 saturation (%)				Yes/No			
Temperature (^{0}C)				Yes/No			
Systolic blood pressure (mmHg)				Yes/No			
Heart rate per minute				Yes/No			
Consciousness				Yes/No			

Oxygenation: NRM ☐ Ventimask ☐ O2-glasses ☐ None ☐

Oxygen in liters per minute: ☐☐

MODIFIED EARLY WARNING SCORE - UMCG							
**Score**	**3**	**2**	**1**	**0**	**1**	**2**	**3**
**Respiratory Rate**		<9		9-14	15-20	21-30	>30
**Heart Rate**		<40	40-50	51-100	101-110	111-130	>130
**Systolic blood Pressure**	<70	70-80	81-100	101-200		>200	
**Consciousness**				A	V	P	U
**Temperature**		<35.1	35.1 - 36.5	36.6 - 37.5	>37.5		
**Saturation**	<90%	<92%	<95%	>95%			
A = Alert	V = Response on voice		P = Response on pain		U = No response		
If production of urine < 75ml in the last 4 hours					Add 1 point		
In case of worries about clinical status of patient					Add 1 point		

Subject nummer ☐☐☐☐☐☐

G03. Preparation by ED

G03-01: Team prepared: Yes ☐ No ☐

G03-02: Preparation: Teamwork and consultation ☐

Preparation of instrumentation ☐

G04. Handover

G04-01: Amount of patients in waiting room: ☐☐

G04-02: Amount of patients on ED: ☐☐

G04-03: Time of entrance ED: ☐☐ ☐☐

G04-04: Time of entrance room: ☐☐ ☐☐

G04-05: Time of arrival nurse : ☐☐ ☐☐

G04-06: Time of arrival physician: ☐☐ ☐☐

G04-07: Time start of handover: ☐☐ ☐☐

G04-08: Time of stop handover: ☐☐ ☐☐

G04-09: Waiting time until handover: ☐☐ minutes

G04-10: Duration of handover: ☐☐ minutes

Subject nummer ☐☐☐☐☐☐

Team ED

G04-11: ED Nurse present: During whole handover ☐ Partly ☐ Absent ☐

If partly ↓

More than half of the time ☐ Less than half of the time ☐

G04-12: Physician present During whole handover ☐ Partly ☐ Absent ☐

If partly ↓

More than half of the time ☐ Less than half of the time ☐

Handover instrument

G04-13 Use of handover instrument:

**ABCDE**		**AMPLE**		**SBAR**	
**A**irway	☐	**A**llergy	☐	**S**ituation	☐
**B**reathing	☐	**M**edication	☐	**B**ackground	☐
**C**irculation	☐	**P**ast	☐	**A**ssessment	☐
**D**isability	☐	**L**ast meal	☐	**R**ecommendation	☐
**E**xposure	☐	**E**vents	☐		
G04-14: Correct order:	☐		☐		☐

G04-15: Ambulance mentioned administered medication: Yes ☐ No ☐

G04-16: Mentioned medication corresponds with Yes ☐ No ☐

pre-hospital information:

G04-17: Mechanism of injury mentioned by ambulance: Yes ☐ No ☐

G04-18: Interruptions during handover : Yes ☐ No ☐

G04-19: Verifying questions asked by ED personell: Yes ☐ No ☐

G04-20: Clarifying questions asked by ED personell: Yes ☐ No ☐

G04-21: Transfer of patient before or after handover: Before ☐ After ☐
