# Supplementary material for: The satisfaction regarding handovers between ambulance and emergency department nurses: an observational study
Source: Scand J Trauma Resusc Emerg Med. 2018 Sep 10;26:78. doi: 10.1186/s13049-018-0545-7 (PMC6131795; doi:10.1186/s13049-018-0545-7)
Supplement: Supplementary file 2 — “Questionnaire”, PDF. The questionnaires used for the measurement of satisfaction regarding the handover. Both teams (handing over and receiving) used a different questionnaire. (PDF 134 kb) [file 13049_2018_545_MOESM2_ESM.pdf]

Additional file 2

Subject nummer | | | | | |

Years of work experience on the ambulance | | |

Score satisfaction of handover

Completed by:
O Ambulance Nurse

I am satisfied with this handover
O Strongly disagree
O Disagree
O Neutral
O Agree
O Strongly agree

Improvement needed regarding this handover
O Composition of ED team
O Timing of handover
O Questions asked by ED team
O Interruptions during handover
O Waiting time too long
O Duration of handover
O Other ……………………………………………………………………………..............

Good factors regarding this handover
O Composition of ED team
O Timing of handover
O Questions asked by ED team
O No interruptions during handover
O Waiting time
O Duration of handover
O ED Team listened carefully
O Other ……………………………………………………………………………..............

Subject nummer | | | | | |

Years of work experience on the ED | | |

Score satisfaction ED personell

Completed by:
O ED nurse
O Physician

I am satisfied with this handover
O Strongly disagree
O Disagree
O Neutral
O Agree
O Strongly agree

Improvement needed regarding this handover
O Pre-hospital information insufficient
O No use of handover instrument (ABCDE, AMPLE, SBAR)
O Incomplete handover
O Timing of handover
O Interruptions during handover
O Waiting time until handover
O Duration of handover
O Other: …………………………………………………………………………….............

Good factors regarding this handover
O Pre-hospital information
O Use of handover instrument (ABCDE, AMPLE, SBAR)
O Complete handover
O Timing of handover
O No interruptions
O Waiting time until handover
O Duration of handover
O Listened carefully
O Other …………………………………………………………………………….............

Correct medical information received ☐

Incorrect medical information received ☐

Diagnosis mentioned ☐

No diagnosis mentioned ☐
